# Bone mineral density and muscle mass associated with healthy eating index in postmenopausal women; results from RaNCD cohort study

**DOI:** 10.1186/s12905-023-02774-6

**Published:** 2023-11-17

**Authors:** Negin Kamari, Hawal Lateef Fateh, Yahya Pasdar, Shahab Rezaeian, Ebrahim Shakiba, Farid Najafi

**Affiliations:** 1 School of Nutritional Sciences and Food Technology, Kermanshah University of Medical Sciences, Kermanshah, Iran; 2 Nursing Department, Kalar Technical College, Garmian Polytechnic University, Kalar, Kurdistan region Iraq; 3 Research Center for Environmental Determinants of Health (RCEDH), Health Institute, Kermanshah University of Medical Sciences, Kermanshah, Iran; 4 Infectious Diseases Research Center, Health Institute, Kermanshah University of Medical Sciences, Kermanshah, Iran; 5 Behavioral Disease Research Center, Kermanshah University of Medical Sciences, Kermanshah, Iran

**Keywords:** Post menopause, Healthy eating index, Bone mineral density, Skeletal muscle

## Abstract

**Background:**

The Healthy Eating Index 2015 (HEI-2015) is a tool for checking the quality of diet. This index is used to examine how well people’s dietary behavior fits certain criteria for achieving a healthy diet in Dietary Guidelines for Americans 2015-2020. We look at the possible association between the HEI-2015 and bone mineral density (BMD) and muscle strength in postmenopausal women.

**Methods:**

This research included 1012 postmenopausal women from the recruitment phase of the Ravansar Noncommunicable Diseases (RaNCD) cohort study in Kermanshah-Iran. A standardized and repeatable food-frequency questionnaire (FFQ) that contains 118 items was used to quantify dietary consumption. Anthropometric indices measured via Bio-Impedance Analyzer BIA (Inbody 770, Inbody Co, Seoul, Korea).

**Result:**

The mean age of postmenopausal women was (56.41 ± 5.31) years. Participants in the highest quartile had a more daily energy intake compared to the lowest quartile (2329.89 ± 837.59), (*P* < 0.001). Women in the upper quartiles had greater Skeletal Muscle Mass (SMM) than those in the lower quartiles (21.61 ± 2.80 vs 20.52 ± 3.13; *p* = 0.0002). The linear model didn’t show a significant relationship between HEI score and greater BMD (*β* = 0.0014, *P* = 0.169).

**Conclusion:**

A diet of high quality followed by a high HEL-2015 score was shown to be strongly connected to increased BMD and muscle mass in Kurdish postmenopausal women.

## Introduction

The postmenopausal phase marks a pivotal period in a woman’s life, characterized by a complex interplay of physiological changes. It is during this life stage that a woman experiences the cessation of menstrual cycles, primarily due to the significant decline in estrogen levels [1]. Postmenopausal women are at an increased risk of conditions such as osteoporosis and muscle loss, conditions that can have profound consequences for their physical well-being, mobility, and overall quality of life [2]. Diet is an important modifiable risk factor for chronic diseases [3]. Furthermore, food can impact BMD and skeletal muscle both before and after menopause [4]. Protein consumption is connected to muscle mass [5], and various studies emphasize the need for enough calcium and vitamin D consumption to avoid osteoporosis and fractures during this life stage [6]. The 2015-2020 Dietary Guidelines for Americans (DGA) indicate a shift away from specific nutrients and foods and toward overall healthy eating patterns, giving nutritional guidelines for every culture and recommending numerous good eating patterns [7, 8]. The Healthy Eating Index (HEI) is a measure that estimates an individual’s diet quality [9], An unhealthy diet, as indicated by lower HEI scores, has been linked to adverse bone outcomes [10–12]. Most evidence related to bone health has been restricted to BMD analysis [1]. studied exclusively in postmenopausal women [12, 13]. also, HEI was revised every 5 years and has been linked to a reduction in the risk of cancer and cardiovascular disease [13, 14]. While the HEI’s most essential characteristics have always been aligned with nutritional advice for bone health [9]. A few research investigations have been conducted yet to investigate the association between adherence to older versions of HEI and various indicators of bone health [15, 16].

Furthermore, adherence to HEI recommendations has been linked to increased lifespan and muscular strength [17]. In addition, various components of the Alternative Healthy Eating Index (AHEI-2010) have been studied in connection to sarcopenia [18]. Accordingly, the purpose of the present research was to assess if a Healthy eating index is related to skeletal muscle and BMD in post-menopausal women living in Kermanshah-Iran.

## Method

### Study design and participants

Data first phase of the Ravansar Non-Communicable Diseases (RaNCD) Cohort investigation was used in this cross-sectional investigation. Ravansar, with a population of 50,000 people, is a district in Kermanshah-Iran. The RaNCD cohort is part of the Prospective Epidemiological Research Studies in Iran (PERSIAN) [19], which began in 2014 with the participation of 10,047 persons aged 35 to 65. The RaNCD research methodology and design were reported in 2019 [20]. From all data in RaNCD, 1672 postmenopausal women satisfied our research requirements. Cancer patients (*n* = 83), diabetics (*n* = 870), and people with high blood pressure (*n* = 1579) were eliminated. Finally, 1012 people were investigated.

### Data collection

Questionnaire information was completed by experts of the cohort center through face-to-face interviews. An online computerized data collection form was used to catch demographic data like age, sex, marital status, socioeconomic status (SES), and smoking. A standard cohort questionnaire was applicable to determine physical activity levels [21]. This questionnaire is self-reported and covers 22 questions on your typical daily sports, work, and leisure activities.

### Anthropometry

Body weight was measured using a bioelectrical impedance analyzer (BIA) Inbody 770, Inbody Co, Seoul, Korea with a precision of 0.5 kg [22]. Other variable such as Muscle Mass (SMM) and Bone Mineral Density (BMD) was also done using BIA. The height of the participants was measured by BSM 370 (Biospace Co, Seoul, Korea) with a precision of 0.1 cm. WC was measured with a flexible measuring tape at the midpoint between the lower rib margin and the iliac crest to the nearest 0.5 cm. BMI was calculated by the following formula: weight (kg) divided by the square of height (m).

### Dietary assessment and healthy eating index 2015

The standardized 137-item 1-year food frequency questionnaire (FFQ) [23] of the PERSIAN cohort study was used to calculate HEI via the Krebs-Smith et al. method [13]. The thirteen ingredients of the HEI 2015 include fruits, whole protein meals, vegetables, seafood and plant proteins, beans, whole grains, milk, lipids, refined grains, salt, added sugars, and saturated fats. Using sufficiency and moderation food categories, these items were split into two groups. The score is greater if the usage is proper. Fruits, vegetables, whole protein meals, seafood, plant proteins, greens and beans, whole grains, milk, and fatty acids should all be a part of a healthy diet. A lower HEI 2015 level, on the other hand, denotes a moderate intake of the last four food groups that make up a balanced diet: refined grains, salt, added sugars, and saturated fats. The first six meals are assessed on a scale of 0 to 5, and the subsequent meals are graded on a scale of 0 to 10. Altogether HEI concession, which ranges from 0 to 100, is the total of the values for any ingredients. The HEI ratings were lowest and greatest in the first and fourth quartiles, respectively (Table 1).

**Table 1:** Healthy eating index—2015^1^

Component	Standard for maximum score^**a**^	Standard for minimum score of zero	Maximum points
Adequacy:			
Total Fruits^b^	≥ 0.8 cup equivalent per 1000 kcal	No Fruit	5
Whole Fruits^c^	≥ 0.4 cup equivalent per 1000 kcal	No Whole Fruit	5
Total Vegetables^d^	≥ 1.1 cup equivalent per 1000 kcal	No Vegetables	5
Greens and Beans^e^	≥ 0.2 cup equivalent per 1000 kcal	No Dark-Green Vegetables or Legumes	5
Whole Grains	≥ 1.5 cup equivalent per 1000 kcal	No Whole Grains	10
Dairy^f^	≥ 1.3 cup equivalent per 1000 kcal	No Dairy	10
Total Protein Foods^d^	≥ 2.5 cup equivalent per 1000 kcal	No Protein Foods	5
Seafood and Plant Proteins^d ,f^	≥ 0.8 cup equivalent per 1000 kcal	No Seafood or Plant Proteins	5
Fatty Acids^g^	(PUFAs+MUFAs)/SFAs≥2.5	(PUFAs + MUFAs)/SFAs ≤1.2	10
Moderation:			
Refined Grains	≤ 1.8 oz. equivalent per 1000	≥ 4.3 oz. equivalent per 1000 kcal	10
Sodium	kcal ≤1.1 g per 1000	≥ 2.0 g per 1000 kcal	10
Added Sugars Saturated	kcal ≤6.5% of energy	≥ 26% of energy	10
Fats	≤ 8% of energy	≥ 16% of energy	10
Total score	–	–	100

^a^Intakes between the minimum and maximum standards are scored proportionately

^b^Includes 100% fruit juice

^c^Includes all forms except juice

^d^Includes legumes (beans and peas)

^e^Includes all milk products, such as fluid milk, yogurt, and cheese, and fortified soy beverages

^f^Includes seafood, nuts, seeds, soy products (other than beverages), and legumes (beans and peas)

^g^Ratio of poly- and mono-unsaturated fatty acids (PUFAs and MUFAs) to saturated fatty acids (SFAs)

### Dietary pattern

e major dietary patterns were identified by principal component analysis to energy-adjusted food intake using data from the RaNCD FFQ. At the beginning, we categorized all food items considering nutrient similarity into 31 food groups (Table 2). In the method of principal component analysis, the varimax rotation was applied to create a distinct and straightforward matrix and kept uncorrelated factor variables called the major pattern. e scree-plot was also drawn to determine the number of matrix components (the major dietary patterns). The typical interpretation of the eigenvalues greater than 1 and the Scree diagram implied that three factors should remain. e extracted factors, dietary patterns, were identified based on recent studies. The factor score of each dietary pattern was computed by calculating the factor load from every group’s dietary intake. Food groups with a factor loading exceeding 0.2 were used to correlate between food groups and the known dietary pattern. Participants individually received a score per pattern based on factor scores and then categorized into tertiles according to dietary model scores.

**Table 2:** Food groupings used in the dietary pattern analyses

Food groups	Dietary components
Leafy vegetables	Cauliflower, lettuce, cucumber, onion, green bean, mushroom, pepper, garlic, turnip, others
Fresh fruits	Melon, watermelon, honeydew melon, plums, prunes, apples, cherries, sour cherries, peaches, nectarine, pear, g, date, grapes, kiwi, pomegranate, strawberry, banana, persimmon, berry, pineapple, oranges, others
Dried fruits	Dried apricots, Dried berries, raisins, and other type dried fruits
Dairy	Milk, yogurt, yogurt drink (doogh), cheese, chocolate milk, crud
Tomato	Tomato
Carotene-rich vegetables	Yellow squash, carrot
Condiments	Condiments
Pickles	Pickles
Legumes	All type beans, peas, lentils, mung bean, soy
Whole grain	Dark breads (Iranian), wheat, barley
Starchy vegetables	Corn, eggplant, green peas, green squash
Vegetable oil	Vegetable oil
Natural juices	All fruit juices
Butter	Butter, margarine, mayonnaise
Olive	Olive and olive oil
Organ meat	Heart, kidney, liver, tongue, brain, o al
Red meat	Beef, lamb, minced meat
Fish	All fish type
Processed meat	Hamburger, sausage, delicatessen meat, pizza
Soft drink	Soft drink
Egg	Egg
Poultry	chicken
Snack	Corn puffs, potato chips, French fries
Sweets and desserts	Cookies, cakes, biscuit, mu ns, pies, chocolates, ice, honey, jam, sugar cubes, sugar, candies, others
Tea and coffee	Tea and coffee
Hydrogenated fat	Hydrogenated fats, animal fats
Salt	salt
Potato	Potato
Refined grain	White breads (lavash, baguettes), noodles, pasta, rice

### Statistical analysis

The STATA software version 14.2 (StataCorp, College Station, TX, USA) was used for data analysis. The significance threshold considered is 0.05. Data were reported as mean (standard deviation), and percentages (frequency) for quantitative and qualitative characteristics, respectively, using a standard BMI cut-off for each sex. The features of the study subjects by the HEI quartiles were examined using one-way analysis of variance (ANOVA) and Chi-square tests for categorical and continuous variables, respectively. Analysis of multiple linear regressions was performed to specify the relationship between BMC and HEI.

## Results

In this study, data from 1012 postmenopausal women were evaluated. According to the healthy eating index quartiles, Table 3 shows the fundamental features of the research subjects. Participants in the first quartile (56.78 ± 5.34 years) are older than those in the fourth quartile (55.91 ± 5.35 years). Of the 362 participants in good SES, 101 (50%) fall into the fourth quartile. BMI, WHR, WC, Skeletal Muscle Mass, and Bone Mineral were all greater among participants in the fourth quartile than they were among subjects in the first quartile. Subjects in the highest quartile had the highest BMI (28.77 ± 4.33 kg/m^2^) than the lowest quartile (26.70 ± 5.11 kg/m^2^), (*P* < 0.001). If we distinguish between quartiles according to skeletal muscle mass, we see that the participants in the fourth quartile were 21.61 ± 2.80 while those in the first quartile were 20.52 ± 3.13 (*P* = 0.0002).

**Table 3:** Background characteristics of the study participants according to HEI category

Characteristics	All	Q1	Q2	*Q3*	*Q4*	*P-*value *
Age (years)	56.41 ± 5.31	56.78 ± 5.34	56.43 ± 5.36	56.22 ± 5.12	55.91 ± 5.35	0.292^**^
Residency, n (%)						
Urban	508(50.20)	102 (28.73)	117 (46.70)	128 (64.00)	159 (78.33)	< 0.001
Rural	504(49.80)	253 (71.27)	133 (53.20)	72 (36.00)	44 (21.67)	
Marital status, n (%)						
Married	802(79.25)	275 (77.46)	202 (80.80)	173 (86.50)	149 (73.40)	0.060
Single	7(0.69)	2 (0.56)	1 (0.40)	3 (1.50)	1 (0.49)	
Divorced and other	203(20.06)	80 (21.98)	47 (18.80)	24 (12.00)	53 (26.11)	
Socio-economic status, n (%)						
Weak	410(40.55)	194 (54.65)	100 (40.00)	59 (29.50)	57 (27.23)	< 0.001
Moderate	239(23.64)	78 (21.97)	60 (24.00)	55 (27.50)	46 (22.77)	
Good	362(35.81)	88 (23.37)	90 (36.00)	86 (43.00)	101 (50.00)	
Physical activity (Met h/day)						
Low	283(27.96)	97 (27.32)	67 (26.80)	64 (32.00)	54 (26.60)	0.091^**^
Moderate	595(58.79)	200 (56.34)	146 (58.40)	118 (59.00)	130 (64.04)	
High	134(13.24)	58 (16.34)	37 (14.80)	18 (9.00)	19 (9.36)	
Current smoker, n (%)	37(3.70)	21 (5.93)	7 (2.87)	4 (2.01)	5 (2.48)	0.282^**^
Alcohol use, n (%)	0	0	0	0	0	
Body Mass Index, kg/m^2^	27.59 ± 4.84	26.70 ± 5.11	27.27 ± 4.66	28.30 ± 4.72	28.77 ± 4.33	< 0.001**
Waist Hip Ratio	0.94 ± 0.059	0.93 ± 0.65	0.94 ± 0.055	0.95 ± 0.055	0.95 ± 0.055	< 0.001
Waist Circumference, cm	98.03 ± 10.92	96.61 ± 12.21	97.81 ± 9.56	99.13 ± 10.96	99.66 ± 9.86	0.005
Skeletal Muscle Mass, kg	20.90 ± 2.90	20.52 ± 3.13	20.75 ± 2.67	21.02 ± 2.72	21.61 ± 2.80	0.0002
Bone Mineral	2.12 ± 0.25	2.09 ± 0.27	2.11 ± 0.24	2.12 ± 0.24	2.18 ± 0.25	0.0009
Hormone replacement therapy	5 (0.50)	0 (0)	1 (0.4)	2 (1.0)	2 (1.0)	0.280^**^

Data are shown mean ± SD for continuous variables and n (%) categorical variables

*P- value was obtained one way anova and Chi square test.

**Variables of age, smoking status, BMI, physical activity and hormone replacement therapy were entered into the multiple model as confounder variables

Table 4 provides information about the dietary intake of postmenopausal women divided by Healthy Eating concessions among the research participants. Regarding daily calorie intake, fourth quartile participants received a higher daily calorie intake (2329.89 ± 837.59) than first quartile participants (2225 ± 709.48) (P < 0.001). If we look at the amount of cholesterol intake, again we see that the participants in the last quartile received a higher daily amount (224.073 ± 116.43) than the participants in the first quartile (202.62 ± 107.41) (*P* = 0.030). Similar to Cholesterol and daily calories, vegetables, Leafy vegetables, fruit, dairy, Nuts, and fish, Olives received higher amounts in the fourth quarter than in the first quarter.

**Table 4:** Dietary intake of postmenopausal women stratified by Healthy Eating score

Variables	Healthy Eating Index score				
	Q1	Q2	Q3	Q4	*P-*value*
**Energy**					
Energy intake (kcal/d)^**^	2225 ± 709.48	2137.57 ± 731.85	2334.95 ± 772.31	2329.89 ± 837.59	< 0.001
Carbohydrate (%E)	60.99 ± 6.97	61.01 ± 6.36	62.15 ± 5.98	62.98 ± 6.30	0.0014
Protein (%E)	12.57 ± 2.08	13.12 ± 2.01	13.58 ± 2.04	14.16 ± 2.30	< 0.001
Fat/oil (%E)	27.92 ± 6.84	27.88 ± 6.58	26.73 ± 5.61	26.25 ± 5.63	0.0057
**Fat/ Oil**					
Saturated fatty acids (g/d)	26.00 ± 12.68	26.16 ± 13.07	25.84 ± 12.45	23.05 ± 12.23	0.030
*Trans fatty acids* (g/d)	0.164 ± 0.247	0.196 ± 0.309	0.243 ± 0.331	0.287 ± 0.377	0.0001
n-3 polyunsaturated fatty acids (mg/d)	0.023 ± 0.020	0.030 ± 0.025	0.034 ± 0.024	0.051 ± 0.044	< 0.001
n-6 polyunsaturated fatty acids (mg/d)	2.43 ± 2.16	3.40 ± 2.64	4.68 ± 3.33	5.79 ± 3.39	< 0.001
Cholesterol (mg/d)	202.62 ± 107.41	215.60 ± 117.78	230.36 ± 121.45	224.073 ± 116.43	0.030
**Food Group**					
Red meat (g/d)	10.59 ± 14.28	14.82 ± 23.77	15.62 ± 16.12	16.71 ± 16.56	0.0002
Processed Meat (g/d)	0.44 ± 1.72	0.93 ± 3.71	0.78 ± 3.10	1.38 ± 4.43	0.0091
Vegetable (g/d)	2.38 ± 4.54	4.21 ± 5.69	7.07 ± 7.72	9.69 ± 7.25	< 0.001
Leafy vegetable (g/d)	149.99 ± 128.63	197.19 ± 153.28	231.08 ± 147	271.70 ± 181	< 0.001
fruit(g/d)	103.90 ± 101	199.74 ± 172	282.296 ± 216	396.503 ± 274	< 0.001
dairy(g/d)	371.53 ± 379	374.98 ± 336	477.078 ± 347	448.134 ± 340	0.0012
Nuts(g/d)	3.35 ± 5.51	5.47 ± 6.17	7.88 ± 9.90	11.69 ± 11.12	< 0.001
fish(g/d)	2.32 ± 4.88	4.11 ± 7.97	4.78 ± 7.57	10.39 ± 15.27	< 0.001
Olive(g/d)	0.121 ± 0.77	0.289 ± 1.16	0.361 ± 1.20	0.559 ± 1.67	0.0004
Refined grain(g/d)	404.50 ± 176	405.92 ± 168	435.50 ± 186	380.97 ± 181	0.023
Whole grain(g/d)	6.11 ± 6.81	9.52 ± 11.51	11.35 ± 11.35	17.15 ± 17.81	< 0.001
Salt(g/d)	4.65 ± 2.60	4.08 ± 2.56	3.75 ± 2.26	3.18 ± 2.06	< 0.001
Vitamin and Mineral^**^					
Vitamin A (mg/d)	4994.82 ± 3202	7016.83 ± 4246	8480.80 ± 4601	11,098.099 ± 7344	< 0.001
Vitamin D (mg/d)	21.96 ± 19.54	28.95 ± 25.42	32.30 ± 2639	46.31 ± 41.76	< 0.001
Vitamin K (μg/d)	107.50 ± 90.56	156.98 ± 108.80	205.03 ± 159.62	248.132 ± 162.35	< 0.001
Vitamin C (mg/d)	61.98 ± 40.12	89.61 ± 57.01	112.47 ± 59.30	148.90 ± 91.04	< 0.001
B12(mg/d)	4.59 ± 3.34	5.63 ± 4.30	5.92 ± 3.63	6.20 ± 3.75	< 0.001
Folate(μg/d)	465.36 ± 181	488.90 ± 188	539.53 ± 186	550.28 ± 225	< 0.001
Calcium(mg)	1026.46 ± 483	1054.23 ± 437	1182.57 ± 432	1132.66 ± 472	0.0005
Magnesium (mg)	236.07 ± 88	274.16 ± 98.72	319.06 ± 111	359.105 ± 139	< 0.001

*P- value was obtained one way anova

** Variables of energy intake and vitamin and mineral use were entered into the multiple model as confounder variables

**Fig. 1:**
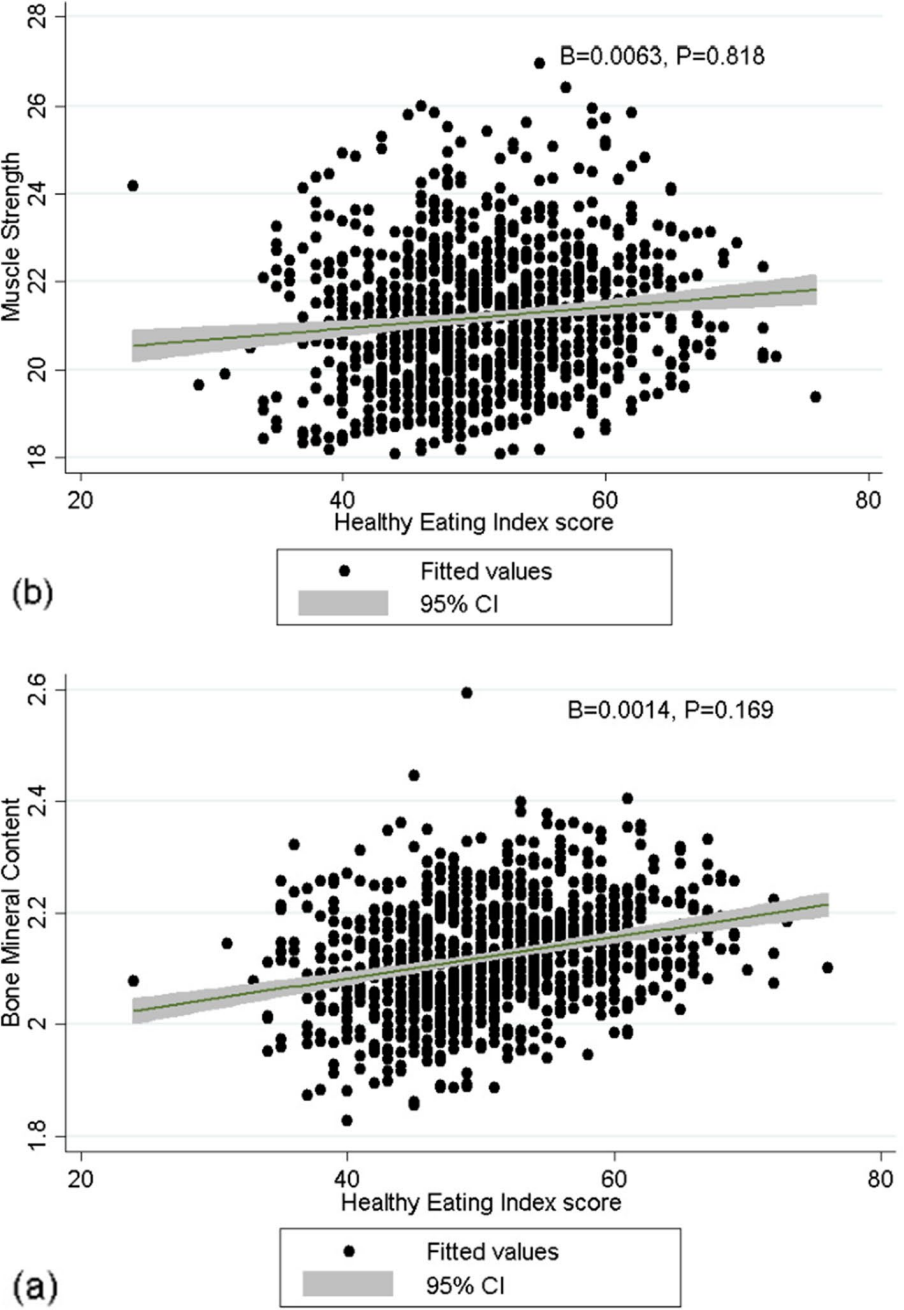
Scatterplot of healthy eating index score on bone mineral content (**a**) and muscle strength (**b**) with adjusting for age, smoking status, BMI, energy intake, physical activity and use of vitamin and mineral complement use

After adjusting for potential confounding factors (age, smoking status, BMI, energy intake, physical activity, and use of vitamin and mineral complement use) in the multiple linear regression analysis, higher HEI score was not associated with greater skeletal muscle mass (*β* = 0.000, *P* = 0.169), greater BMC (*β* = 0.001, *P* = 0.939) and greater muscle strength (*β* = 0.0063, *P* = 0.818) (Fig. 1**).**

## Discussion

To the best of our knowledge, this investigation is the first investigation into the relationship between Kurdish women’s HEI-2015 adherence and their bone health and muscle mass. The results demonstrated that as quartiles of HEI climbed, menopausal women’s bone mineral density and muscle mass dramatically increased. Previous research on potential connections between HEI and bone health has shown conflicting findings [13, 24–26]. In this regard, some studies identified a significant link between HEI compliance and higher BMD [24, 25], while other studies found no such relationship with indications of bone turnover or a higher risk of hip fractures [13, 26].

In this regard, increased BMD in our study was linked to stronger adherence to HEI 2015, while this association was not significant after adjusting for potential confounding factors (Age, Smoking status, BMI, Energy intake, physical activity, and use of vit and mineral complement use). Currently, the comparison of the findings is not clear, since we are not aware of any prior research that looked into the potential association between HEI-2015 and bone mineral density. But Movassaghi and Vatanparast’s examination of 49 studies on the association between dietary type and different bone outcomes, defined retrospectively or a priori [10], which shows that a healthy diet can elevate BMD and thereby lower osteoporosis prevalence, supports our findings. The findings of this study also support Denova-Gutiérrez et al.’s examination that looked at the association between diet and bone health [27], which suggests an adverse relationship between healthy eating with HEI-2015-like components and the risk of low bone density. Additionally, our findings consist of 20 research on the connection between diet quality and bone health, a systematic review and meta-analysis by Fabian et al. [28], and another relevant research demonstrating that a healthy diet like HEI-2015 can diminish the risk of low BMD and fracture by 18 and 41%, respectively.

In our study, we found that there was a significant difference in daily calorie intake and cholesterol intake among participants in different quartiles. Specifically, the fourth quartile participants had a significantly higher daily calorie intake (2329.89 ± 837.59) compared to those in the first quartile (2225 ± 709.48) with a *p*-value of less than 0.001. This finding aligns with a study conducted by de Dues et al. (2016), where they observed a similar trend in daily calorie intake, with participants in the higher quartiles consuming more calories than those in the lower quartiles [29]. Additionally, when it comes to cholesterol intake, our study also showed that participants in the last quartile had a higher daily cholesterol intake (224.073 ± 116.43) compared to those in the first quartile (202.62 ± 107.41) with a p-value of 0.030, a result consistent with the findings of Xu Z et al. (2018) [30]. Furthermore, similar trends were observed in the consumption of various food groups like vegetables, leafy vegetables, fruits, dairy, nuts, fish, and olives, with the fourth quartile participants consistently consuming higher amounts compared to the first quartile, which corresponds with the outcomes of previous research conducted by Khorasani S et al. (2020) [31].

These consistent findings across different studies suggest a robust association between quartile-based dietary patterns and specific nutrient intake.

The essential properties of this score help to explain the substantial link between HEI-2015 increased and higher BMD in this study. Protein, vitamins D, K, and C, calcium, magnesium, zinc, manganese, and potassium make up the most important nutrients in the maintenance of bone health, with established effects on bone structure and/or digestion, and their consumption linked to higher BMD and also decrease the risk of fractures [32–34]. Furthermore, even with adequate calcium consumption, a higher dietary alkaline load from increased magnesium and potassium intake may diminish osteoclasts. There is also growing evidence that it lowers calcium flow to the bone via inhibiting and activating osteoblasts can increase BMD and lower the risk of fractures [35].

Furthermore, increased dietary intake of powerful antioxidant components like vitamins E and C shown to play an important role in fighting bone loss caused by oxidative stress and lowering the risk of osteoporosis [34–36]. Furthermore, increasing the consumption of anti-inflammatory foods like fiber, omega-3, and vitamins D, E, and C reduces inflammation and lowers the risk of low bone mineral density and fractures [37]. Omega-3 PUFA and MUFAs may potentially benefit bone metabolism by decreasing osteoclast action or boosting osteoblastic action, lowering the risk of osteoporosis [38]. Furthermore, folate and vitamin B12 can decrease the incidence of low BMD and fractures, mostly by reducing the damaging influence of hyperhomocysteinemia on bone health [39].

Increased intake of simple sugars may increase the risk of low BMD and fracture by processes such as increased inflammation and hyperinsulinemia, as well as increased renal acid load and urine calcium secretion [35]. On the other hand, excessive salt consumption is linked to an elevated risk of osteoporosis due to urine calcium excretion [30]. Excess SFA intake can also increase the risk of bone remodeling mostly via boosting pro-inflammatory mechanisms and decreasing absorption of calcium in the intestine leading to osteoporosis [30].

Another conclusion from our research is that women in the top tertile of HEI concession had more muscle mass than those in the lowest tertile. Following our findings, Esmaeily et al. [40] showed that holding the HEI-2015 increases muscular strength in elderly adults. Furthermore, Chan et al. demonstrated that higher Diet Quality Index-International (DQI-I), “vegetable-fruit” dietary pattern scores, as well as higher scores for the “foods-beverages-dairy” diet [41] and Mediterranean diet [42], were related to decreasing the risk of sarcopenia. In contrast to previous findings, a cohort study of 757 people in Newcastle discovered a link between a “traditional British” diet strong in butter, red meat, gravy, and potatoes with sarcopenia [43].

However, the precise methods by which a good diet might improve muscle mass are unknown, but several suggestions exist. First, consuming these diets reduces oxidative stress [44]. Oxidative stress stimulates the gene production of inflammatory cytokines such as interleukin-1 (IL-1) and tumor necrosis factor (TNF), that can damage muscle tissue [45]. SFA reduction was also linked to a decreased incidence of sarcopenia [46]. Second, in healthy diets, low salt levels are associated with fat growth and muscular weakening [47].

Furthermore, earlier research has found a link between milk consumption and the risk of sarcopenia [48]. Individuals in the top tertile of HEI consume more dairy products than those in the lowest tertile in the present population (*P* = 0.0012).

## Conclusion

As a result, our study showed that higher adherence to a healthy diet as HEI affect positively BMD and skeletal muscle in a sample of Kurdish menopausal women. In this regard, we can suggest changing dietary patterns as non-pharmacology therapy for the prevention of bone and muscle loss in this period.

Various limitations to this study should be considered. A causal link cannot be established due to the cross-sectional form. Due to a lack of data, some HEI-2015 components, such as alcohol intake, are not included in the HEI-2015 score.

## Data Availability

The data analyzed in the study are available from the corresponding author upon reasonable request.
